# Prognostic impact of restrictive ventilatory defects in chronic lung allograft dysfunction without restrictive allograft syndrome-like opacities: Stratification of emerging undefined and unclassified phenotypes

**DOI:** 10.1016/j.jhlto.2025.100445

**Published:** 2025-11-24

**Authors:** Taiki Fukuda, Yusei Nakamura, Yuki Ko, Shu-Chi Tseng, Staci M. Gagne, Takeshi Johkoh, Yi Li, David C. Christiani, Hiroya Ojiri, Lynette Sholl, Mizuki Nishino, Hiroto Hatabu

**Affiliations:** a Center for Pulmonary Functional Imaging, Department of Radiology, Brigham and Women’s Hospital and Harvard Medical School, Boston, MA; b Department of Radiology, The Jikei University School of Medicine, Tokyo, Japan; c Department of Clinical Radiology, Graduate School of Medical Sciences, Kyushu University, Fukuoka, Japan; d Department of Medical Imaging and Intervention, Chang Gung Memorial Hospital at Linkou and Chang Gung University, Taoyuan City, Taiwan; e Department of Imaging, Dana-Farber Cancer Institute, Boston, MA; f Department of Radiology, Kansai Rosai Hospital, Amagasaki, Hyogo, Japan; g Department of Biostatistics, University of Michigan, Ann Arbor, MI; h Division of Pulmonary and Critical Care Medicine, Department of Medicine, Massachusetts General Hospital and Harvard Medical School, Boston, MA; i Department of Environmental Health, Harvard TH Chan School of Public Health, Boston, MA; j Department of Pathology, Brigham and Women’s Hospital and Harvard Medical School, Boston, MA

**Keywords:** Chronic lung allograft dysfunction, Phenotypes, Prognosis, Restrictive, Lung transplantation, Baseline lung allograft dysfunction, Pleural effusion

## Abstract

**Background:**

Chronic lung allograft dysfunction (CLAD) remains a critical factor affecting post-lung transplant prognosis. While RAS-like opacities (RLO) are established as poor prognostic indicators, prognostic stratification of non-RLO CLAD cases, including undefined and unclassified phenotypes per the 2019 International Society for Heart and Lung Transplantation (ISHLT) criteria, remains unexplored.

**Methods:**

We retrospectively analyzed 241 bilateral lung transplant recipients between 2005 and 2021. CLAD was diagnosed and classified per the 2019 ISHLT criteria. Non-RLO patients were stratified by restrictive ventilatory defects for survival analysis. Diaphragmatic elevation and pleural effusion were also evaluated as potential causes of restrictive physiology and their prognostic significance.

**Results:**

Of 83 CLAD patients, 61 (73.5%) had no RLO. In multivariable Cox analysis of non-RLO patients, restrictive ventilatory defects showed a trend toward worse outcomes (Hazard ratio [HR]: 2.56, 95% CI: 0.98–6.71; *p* = 0.055), while obstructive defects showed no association (HR: 0.88, *p* = 0.785). When accounting for retransplantation as a competing event using Fine-Gray regression, restrictive ventilatory defects were significantly associated with increased cumulative incidence of death (subdistribution HR: 2.96, 95% CI: 1.17–7.51; *p* = 0.022). Pleural effusion showed a trend toward association with restrictive defects (*p* = 0.063) and emerged as an independent prognostic factor (HR: 5.06, 95% CI: 1.57–16.27; *p* = 0.007).

**Conclusions:**

Non-RLO CLAD patients with restrictive ventilatory defects demonstrated significantly increased cumulative incidence of death (retransplantation as a competing risk), challenging the assumption that all non-RLO cases follow a favorable BOS-like prognosis. Pleural effusion was identified as a significant independent prognostic marker.

## Introduction

Chronic lung allograft dysfunction (CLAD) remains a critical factor affecting post-lung transplant prognosis, affecting 50% of patients by 5 years post-transplant.^1^, ^2^ Until recently, there had been no unified consensus regarding CLAD classification. However, in 2019, the International Society for Heart and Lung Transplantation (ISHLT) published a consensus document to standardize the nomenclature of CLAD and its clinical phenotypes.^3^

In this consensus, mixed and undefined phenotypes were newly defined. The undefined phenotype is characterized by either obstructive ventilatory defects without restrictive features but with persistent opacities, namely restrictive allograft syndrome-like opacities (RAS-like opacities; RLO), or mixed obstructive and restrictive ventilatory defects without RLO.^3^ Additionally, Levy et al. reported a phenotype that could not be classified into any of the CLAD phenotypes, which was newly defined as "unclassified" and added to the refined ISHLT criteria.^4^

Several studies have attempted to stratify the prognosis of CLAD patients based on clinical features. The presence of RLO and restrictive ventilatory defects is associated with poor outcomes,^4^, ^5^, ^6^, ^7^ while bronchiolitis obliterans syndrome (BOS), lacking both features, has a relatively better prognosis.^4^ Therefore, both pulmonary function tests (PFTs) and imaging findings play critical roles in prognostic assessment. Notably, undefined and unclassified phenotypes account for 22–41% of CLAD cases, with the majority without RLO.^4^, ^8^, ^9^ While those with RLO have poor prognoses comparable to RAS and mixed phenotypes,^4^ prognostic stratification for the non-RLO CLAD group, which includes BOS and most undefined/unclassified cases, has not been sufficiently analyzed. Therefore, comprehensive stratification of the non-RLO CLAD group is crucial for establishing optimal treatment strategies.^10^

The current ISHLT classification relies on obstructive parameters when categorizing undefined and unclassified phenotypes; however, whether this adequately stratifies prognosis in non-RLO CLAD remains unclear. Should obstructive parameters alone prove inadequate, alternative measures such as restrictive ventilatory defects warrant investigation.

Therefore, the primary aim of this study is to determine whether restrictive ventilatory defects stratify prognosis of the non-RLO CLAD group, including undefined and unclassified phenotypes. As a secondary aim, we sought to identify whether pleural effusion and diaphragmatic elevation, previously reported to cause restrictive physiology,^11^, ^12^ contribute to restrictive ventilatory defects in non-RLO CLAD patients and to assess their prognostic significance. We hypothesized that stratification of the non-RLO CLAD group by the presence or absence of restrictive ventilatory defects would reveal significant differences in prognosis.

## Materials and Methods

### Study design

This study was a single-center retrospective cohort analysis approved by the Mass General Brigham institutional review board (No.2021P002789), with a waiver of informed consent, and in compliance with the ISHLT ethics statement. All lung transplant recipients at our institution between January 2005 and December 2021, based on the pathology database, were included in the initial review. Clinical and radiological data were obtained from medical records. All eligible patients were followed until December 2024. To assess potential selection bias, baseline characteristics were compared between included and excluded patients.

### CLAD definition and phenotype classification

We used the definition of CLAD and its phenotypes per the 2019 ISHLT criteria.^3^ To evaluate CLAD, the following inclusion criteria were used: 1) patients undergoing first bilateral-lung transplantation or heart-lung transplantation; 2) post-transplant survival exceeding 3 months; and 3) availability of sufficient follow-up data for CLAD diagnosis, specifically: PFTs data including baseline post-transplant measurements and serial follow-up measurements for CLAD diagnosis; and persistent opacities, namely RLO, documented on chest imaging (CT and/or chest x-ray [CXR]) at the time of CLAD diagnosis. We defined RLO as ground-glass opacities, consolidation, or reticular opacities and/or progressive pleural thickening, suggesting fibrosis on chest imaging.^3^, ^4^ For restrictive ventilatory defects, while TLC ≤ 90% compared to baseline is the gold standard per the 2019 ISHLT consensus,^3^ we used FVC ≤ 80% compared to baseline as a surrogate marker when TLC was unavailable, as recommended.^4^, ^13^ This FVC threshold has been validated in multiple independent cohorts as a reliable predictor of restrictive physiology and adverse outcomes in CLAD patients.^6^, ^7^, ^14^ Pulmonary function testing was performed by spirometry according to the American Thoracic Society/European Respiratory Society (ATS/ERS) standardized guidelines.^15^

### Image acquisition and interpretation

Two thoracic radiologists, with 5- and 12-year experiences, respectively, independently reviewed all chest images. Any discrepancies were resolved by consensus with a third radiologist (with more than 20 years of experience). CXRs were obtained in the posteroanterior (PA) view during adequate inspiration. CT images were reconstructed with 1-mm slice thickness and displayed at lung window settings (level, −600 Hounsfield unit; width, 1500 Hounsfield unit) on inspiration.

### Assessment of diaphragmatic elevation

Diaphragmatic elevation was evaluated at the time of CLAD diagnosis using CXR, as it is one of the most commonly used diagnostic tools for the initial evaluation of suspected diaphragmatic dysfunction.^16^, ^17^ We examined PA view CXRs taken on the date closest to CLAD diagnosis. Additionally, follow-up CXRs obtained at least 2 months later were also evaluated to confirm the presence of diaphragmatic elevation. Diaphragmatic elevation was defined as follows: a right-sided dysfunction is present when the right hemidiaphragm is > 4 cm higher than the left side, whereas left-sided dysfunction is present when the left hemidiaphragm is at the same height or higher than the right side.^17^ Note that this methodology allowed for the detection of unilateral but not bilateral diaphragm elevation.

### Assessment of pleural effusion

Pleural effusion was assessed using CT when available at CLAD diagnosis and/or follow-up, with any visible fluid attenuation classified as effusion present. When CT was unavailable, PA view CXR was used with effusion identified by costophrenic angle blunting.^18^ Follow-up imaging at least 2 months later confirmed persistence and excluded transient effusions. Effusions were classified as present or absent regardless of laterality or volume. Clinical workup was performed to identify the underlying etiology and exclude acute reversible causes. This included a review of medical records for documented comorbid conditions. Cases with pleural effusion met CLAD diagnostic criteria if a persistent ≥20% FEV1 decline was observed for ≥3 months either after appropriate treatment,^3^ or when effusion was minimal with no identifiable cause requiring no treatment.

### Assessment of baseline radiographic findings

To evaluate temporal relationships between radiographic findings and CLAD development, baseline imaging was retrospectively reviewed in non-RLO CLAD patients with pleural effusion or diaphragmatic elevation. Baseline images were defined as those obtained at the time point corresponding to baseline FEV1 calculation (mean of best two post-operative FEV1 values >3 weeks apart).^3^ When multiple images were available, the image closest to the midpoint of the two FEV1 measurements was selected. Radiographic findings were assessed using the same methodology described above, with CT preferentially used for pleural effusion and CXR for diaphragmatic elevation. Findings were classified as persistent (present at both timepoints) or new-onset (absent at baseline, present at CLAD diagnosis), with trajectories evaluated through interval imaging review.

### Statistical analysis

Categorical variables were presented as counts and percentages, and continuous variables were presented as medians with interquartile ranges (IQRs), as appropriate. Mann-Whitney U test and Kruskal-Wallis test were used for continuous variable comparisons, while chi-square test and Fisher's exact test were used for categorical variables. Binary logistic regression was used to further investigate associations between categorical variables. Overall survival (OS) was analyzed using Kaplan-Meier curves and log-rank test. In the primary analysis, OS included both death and retransplantation as events, measured in months from CLAD diagnosis, with patients censored at the end of the study. Multivariable Cox proportional hazard models were performed, with age and primary disease included a priori based on their established associations with post-transplant survival,^19^, ^20^ except in models with multiple clinical variables, where limited event numbers required fewer covariates. To assess potential multicollinearity among covariates in multivariable models, variance inflation factors (VIF) were calculated using the variance inflation factor function. VIF values < 5 were considered to indicate acceptable levels of collinearity.^21^ Additionally, to account for retransplantation as a competing event for death, competing risks regression was performed using the Fine-Gray model as a complementary analysis, with subdistribution hazard ratios (SHR) estimated for all multivariable models. Interobserver agreement for the assessment of diaphragmatic elevation and pleural effusion was evaluated using Cohen's kappa coefficient. Kappa values were interpreted using standard criteria.^22^ Median follow-up time was calculated by the reverse Kaplan-Meier method.^23^ Statistical analyses were performed using SPSS version 30.0 (IBM Corp., Armonk, NY) and R software version 4.5.0 (R Foundation for Statistical Computing, Vienna, Austria), with *p*-values < 0.05 considered significant.

## Results

### Characteristics of patients with CLAD

**Figure 1:**
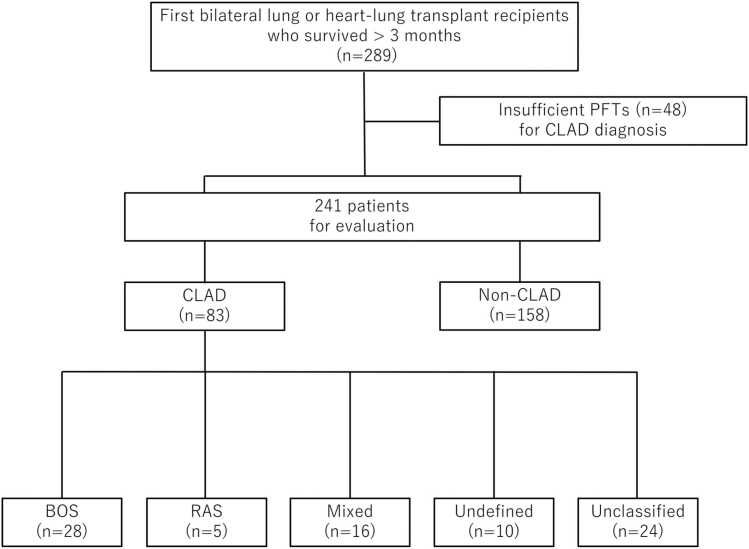
Flow diagram of the study cohort. BOS, bronchiolitis obliterans syndrome; CLAD, chronic lung allograft dysfunction; CT, computed tomography; CXR, chest x-ray; PFTs, pulmonary function tests; RAS, restrictive allograft syndrome.

Figure 1 represents the flow diagram of the study cohort. Of 289 first bilateral lung transplant recipients between January 2005 and December 2021, 48 were excluded due to insufficient PFTs data. Baseline characteristics of included and excluded patients are presented in Supplemental Table 1. Among the 241 patients, 83 met the new ISHLT criteria for CLAD and were included. Two patients in our cohort received heart-lung transplantation, but neither developed CLAD during the study period. Of the 83 patients diagnosed with CLAD, chest CT was available at the time of CLAD diagnosis in 67 patients (80.7%), while the remaining 16 patients (19.3%) were assessed using CXR alone.

**Figure 2:**
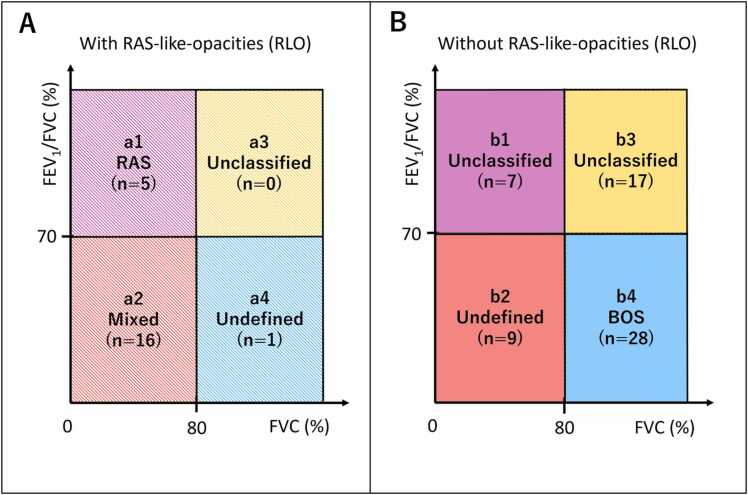
Classification of CLAD phenotypes based on ventilatory patterns and radiographic findings. Cases are stratified by the presence or absence of obstructive defects (FEV1/FVC <70%, vertical axis), restrictive defects (FVC ≤80% predicted, horizontal axis), and RAS-like opacities (RLO). Each quadrant is labeled (a1-a4, b1-b4) with corresponding phenotypes and number of cases. (A) Cases with RLO are classified into RAS, mixed, unclassified, and undefined phenotypes. (B) Cases without RLO are categorized into BOS, undefined, and unclassified phenotypes. BOS, bronchiolitis obliterans syndrome; CLAD, chronic lung allograft dysfunction; FEV₁, forced expiratory volume in one second; FVC, forced vital capacity; RAS, restrictive allograft syndrome; RLO, RAS-like opacities.

CLAD phenotypes included BOS (n=28, 33.7%), RAS (n=5, 6.0%), mixed (n=16, 19.3%), undefined (n=10, 12.0%), and unclassified (n=24, 28.9%). During a median follow-up of 43.7 months (95% confidence interval [CI]: 35.6–51.9), 42 patients either died (n=40) or underwent retransplantation (n=2), with a median time to death or retransplantation of 53.5 months (95% CI: 37.4–69.5). Baseline characteristics are shown in Table 1. Figure 2 illustrates phenotypic distribution based on combinations of obstructive and restrictive ventilatory defects and RLO presence. Only one undefined/unclassified case exhibited RLO.

**Table 1:** Patient Characteristics of CLAD

Characteristics	CLAD (n=83)
Age at Tx, years, median (IQR)	56.4 (47.2-63.7)
Gender male, n (%)	45 (54.2)
Primary disease, n (%)	
Pulmonary fibrosis	44 (53.0)
COPD/emphysema	20 (24.1)
Cystic fibrosis	12 (14.5)
Other	7 (8.4)
Time from Tx to CLAD, months (IQR)	36.0 (21.5-60.5)
CLAD phenotype	
BOS, n (%)	28 (33.7)
RAS, n (%)	5 (6.0)
Mixed, n (%)	16 (19.3)
Undefined, n (%)	10 (12.0)
Unclassified, n (%)	24 (28.9)

Abbreviations: BOS, bronchiolitis obliterans syndrome; CLAD, chronic lung allograft dysfunction; COPD, chronic obstructive pulmonary disease; IQR, interquartile range; RAS, restrictive allograft syndrome; Tx, transplant.

### Survival analysis of undefined, unclassified and BOS phenotypes

Among 62 patients diagnosed with undefined, unclassified and BOS phenotypes, 24 either died (n = 22) or underwent retransplantation (n = 2, of which 1 was in the non-RLO group), with a median time from CLAD diagnosis to death or retransplantation of 67.8 months (95% CI: 53.4-not estimable). The median follow-up duration was 40.3 months (95% CI: 35.0–56.4). No significant survival differences were observed among these three groups (median OS: undefined 41.7 months, unclassified 55.4 months, BOS 68.1 months; *p* = 0.392). In a multivariable Cox proportional hazards model adjusted for age and primary disease, neither undefined (Hazard ratio [HR]: 2.83, 95% CI: 0.82–9.77, *p* = 0.099) nor unclassified phenotypes (HR: 1.45, 95% CI: 0.55–3.81, *p* = 0.455) differed significantly from BOS.

### Survival analysis of ventilatory patterns in non-RLO CLAD patients

Among the 61 non-RLO CLAD patients (Figure 2B: b1-b4), 45 (73.8%) had CT available at CLAD diagnosis, and 16 (26.2%) were assessed by CXR alone. Sixteen patients (26.2%) had restrictive ventilatory defects (b1, b2), and 45 patients (73.8%) did not (b3, b4). Patients with restrictive defects had a median OS of 55.4 months (95% CI: 7.3–103.4) compared to 70.5 months (95% CI: 56.4–84.5) in those without (*p* = 0.127). For obstructive defects (b2, b4 vs b1, b3), patients with defects had a median OS of 68.1 months (95% CI: 58.0–78.3) compared to 55.4 months (95% CI: 27.6–83.1) in those without (*p* = 0.840). In a multivariable Cox proportional hazards model adjusted for age and primary disease, the HR for restrictive ventilatory defects was 2.56 (95% CI: 0.98–6.71; *p* = 0.055) and for obstructive ventilatory defects was 0.88 (95% CI: 0.36–2.15; *p* = 0.785). When accounting for retransplantation as a competing event using Fine-Gray regression, restrictive ventilatory defects were significantly associated with increased cumulative incidence of death (SHR: 2.96, 95% CI: 1.17–7.51; *p* = 0.022), while obstructive defects showed no significant association (SHR: 0.70, 95% CI: 0.28–1.72; *p* = 0.438).

### Survival analysis of CLAD stratified by RLO status and restrictive ventilatory defects

**Figure 3:**
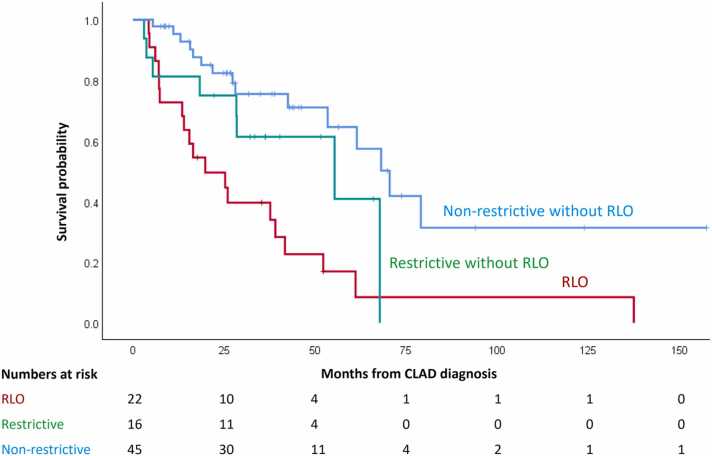
Survival analysis of CLAD patients stratified by RLO status and restrictive ventilatory defects. Kaplan-Meier survival curves demonstrated statistically significant differences in overall survival from the time of CLAD diagnosis among these groups (*p* < 0.001). Patients with RLO showed the poorest prognosis, while non-RLO CLAD patients without restrictive ventilatory defects demonstrated the most favorable outcomes. CLAD, chronic lung allograft dysfunction; RAS, restrictive allograft syndrome; RLO, RAS-like opacities.

To further explore the combined impact of RLO status and restrictive physiology, we stratified patients into three groups: those with RLO (Figure 2A: a1-a4), those without RLO but with restrictive ventilatory defects (Figure 2B: b1, b2), and those without either feature (b3, b4). Patient demographic and clinical characteristics are shown in Table 2. Kaplan-Meier analysis showed significant survival differences among these groups (*p* < 0.001). The RLO group had the poorest outcomes (median OS: 19.8 months; 95% CI: 5.5–34.2), followed by the non-RLO CLAD with restrictive ventilatory defects group, while the non-RLO CLAD without restrictive ventilatory defects group demonstrated the most favorable prognosis (median OS: 70.5 months; 95% CI: 56.5–84.5) (Figure 3).

**Table 2:** Patient Characteristics of RLO, Non-RLO with/without Restrictive Defects

Characteristics	RLO	Non-RLO with Restrictive	Non-RLO without Restrictive	*p* -value
Number of patients, n (%)	22 (26.5)	16 (19.3)	45 (54.2)	
Age at Tx, years, median (IQR)	52.1 (41.0-57.0)	57.9 (50.4-64.1)	57.9 (52.7-64.6)	0.074
Gender male, n (%)	11 (50.0)	5 (31.2)	29 (64.4)	0.065
Primary disease, n (%)				0.183
Pulmonary fibrosis	13 (59.1)	10 (62.5)	21 (46.7)	
COPD/emphysema	2 (9.1)	3 (18.8)	15 (33.3)	
Cystic fibrosis	5 (22.7)	3 (18.8)	4 (8.9)	
Other	2 (9.1)	0	5 (11.1)	
Time from Tx to CLAD months (IQR)	28.5 (21.2-46.8)	35.0 (19.8-72.5)	39.0 (28.0-61.0)	0.438

Abbreviations: BOS, bronchiolitis obliterans syndrome; CLAD, chronic lung allograft dysfunction; COPD, chronic obstructive pulmonary disease; IQR, interquartile range; RAS, restrictive allograft syndrome; RLO, RAS-like opacities; Tx, transplant.

In the multivariable Cox proportional hazards model adjusted for age and primary disease, the RLO group showed significantly worse prognosis (HR: 5.50, 95% CI: 2.43–12.47, *p* < 0.001), while non-RLO CLAD patients with restrictive ventilatory defects demonstrated a non-significant trend toward worse outcomes (HR: 2.48, 95% CI: 0.98–6.27, *p* = 0.055). To account for retransplantation as a competing event, we performed Fine-Gray competing risks regression analysis. In this analysis, both the RLO group (SHR: 5.26, 95% CI: 2.58–10.74, *p* < 0.001) and non-RLO CLAD patients with restrictive ventilatory defects (SHR: 2.60, 95% CI: 1.08–6.24, *p* = 0.033) were significantly associated with increased cumulative incidence of death (Table 3).

**Table 3:** Multivariable Fine-Gray Competing Risk Models Evaluating Clinical Variables Across CLAD Patients Stratified by RLO Status And Restrictive Ventilatory Defects With Time From CLAD Diagnosis to Death, with Retransplantation as a Competing Event

Variables	Hazard Ratio	95% CI	*p* -value
RLO	5.26	2.58-10.74	< 0.001
Non-RLO with restrictive	2.60	1.08-6.24	0.033

Abbreviations: CI, confidence intervals; CLAD, chronic lung allograft dysfunction; RAS, restrictive allograft syndrome; RLO, RAS-like opacities.

Non-RLO CLAD patients without restrictive ventilatory defects were used as the reference category. Adjusted for age at CLAD diagnosis and primary disease. Primary disease categorized into chronic obstructive pulmonary disease/emphysema, pulmonary fibrosis, and other.

### Radiographic findings of pleural effusion and diaphragmatic elevation in non-RLO CLAD patients

**Figure 4:**
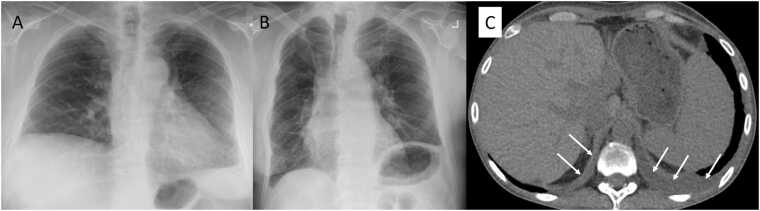
Representative images of diaphragmatic elevation and pleural effusion in patients with restrictive ventilatory defects without RLO. (A) CXR image demonstrating right hemidiaphragm elevation of more than 4 cm higher than the left hemidiaphragm, with a patient still alive at 33 months after CLAD diagnosis. (B) CXR image demonstrating left hemidiaphragm elevation higher than the right hemidiaphragm, with a patient still alive at 32 months after CLAD diagnosis. (C) CT image showing predominantly left-sided pleural effusion (white arrows). The patient died 28 months after CLAD diagnosis. CLAD, chronic lung allograft dysfunction; CT, computed tomography; CXR, chest x-ray; RAS, restrictive allograft syndrome; RLO, RAS-like opacities.

Interobserver agreement for the assessment of radiographic findings demonstrated good agreement for both pleural effusion (κ = 0.71) and diaphragmatic elevation (κ = 0.61). Among 61 non-RLO CLAD patients (Figure 2B: b1-b4), CT was available at CLAD diagnosis and/or follow-up for pleural effusion assessment in 58 patients (95.1%), while the remaining 3 patients (4.9%) were evaluated by CXR alone. Using these imaging modalities, twelve patients (19.7%) were found to have pleural effusions. Etiological evaluation identified chronic heart failure (n=1) and chronic kidney disease (n=3), while 8 patients had no identifiable cause despite comprehensive workup. Three patients received therapeutic interventions (thoracentesis with diuretics, n=1; diuretics alone, n=2), all initiated at or before CLAD diagnosis. The remaining 9 patients received no treatment due to minimal effusion and clinical stability. Diaphragmatic elevation was present in 29 patients (47.5%). Representative images of diaphragmatic elevation and pleural effusion are shown in Figure 4.

### Temporal characteristics of pleural effusion and diaphragmatic elevation

Baseline imaging was reviewed to determine whether radiographic findings at CLAD diagnosis were pre-existing or new-onset. For pleural effusion (n=12), baseline CT was available in 9 patients (75.0%) and CXR in all 12 patients. The majority (11/12, 91.7%) showed persistent effusion from baseline, with only one new-onset case developing in the context of chronic kidney disease prior to CLAD diagnosis. For diaphragmatic elevation (n=29), baseline CXR was available in all patients. Most (26/29, 89.7%) had persistent elevation from baseline, while three (10.3%) developed new-onset elevation that gradually became apparent between baseline and CLAD diagnosis.

### Association between radiographic findings and restrictive ventilatory defects

In univariate analysis, the associations between restrictive ventilatory defects (Figure 2B: b1, b2) and both diaphragmatic elevation (*p* = 0.163) and pleural effusion (*p* = 0.063) did not reach statistical significance. Binary logistic regression analysis, including pleural effusion, diaphragmatic elevation, and obstructive ventilatory defects, revealed that pleural effusion had the strongest association with restrictive ventilatory defects (odds ratio: 3.72; 95% CI: 0.82–16.88; *p* = 0.089), though this association did not reach statistical significance.

### Survival analysis of radiographic findings in non-RLO CLAD patients

When analyzing the entire non-RLO CLAD (Figure 2B: b1-b4) cohort using the Kaplan-Meier method, patients with pleural effusion had significantly shorter median OS compared to those without (median OS: 21.9 months; 95% CI: 8.2–35.5 vs 68.1 months; 95% CI: 64.0–72.3; *p* < 0.001). In contrast, diaphragmatic elevation showed no significant difference in OS between patients with and without elevation (median OS: 55.4 months; 95% CI: 20.4–90.3 vs 70.5 months; 95% CI: 67.0–74.0; *p* = 0.248). In a multivariable Cox proportional hazards model adjusted for age and primary disease, pleural effusion was significantly associated with poor prognosis (HR: 5.41, 95% CI: 2.04–14.39, *p* < 0.001), while diaphragmatic elevation showed no significant association (HR: 1.69, 95% CI: 0.73–3.95, *p* = 0.224). When accounting for retransplantation as a competing event using Fine-Gray regression, the prognostic associations remained consistent, with pleural effusion showing a significant association (SHR: 6.72, 95% CI: 2.71–16.65, *p* < 0.001) and diaphragmatic elevation showing no significant association (SHR: 2.00, 95% CI: 0.87–4.59, *p* = 0.101).

To further explore the relationships between ventilatory patterns and radiographic findings, we conducted a comprehensive multivariable analysis. In a Cox proportional hazards model, including restrictive (b1, b2) and obstructive ventilatory defects (b2, b4), pleural effusion, and diaphragmatic elevation, pleural effusion was significantly associated with increased risk of death/retransplantation (HR, 5.06; 95% CI: 1.57–16.27; *p* = 0.007), while the association between restrictive ventilatory defects and prognosis was attenuated (HR, 1.27; 95% CI: 0.45–3.54; *p* = 0.650) (Table 4). Assessment of multicollinearity using VIF revealed no substantial collinearity among these covariates, with VIF values ranging from 1.27 to 1.62 (all < 5).

**Table 4:** Multivariable Cox Proportional Hazards Models Evaluating Four Clinical Variables Across Non-RLO CLAD and Their Association With Time From CLAD Diagnosis To Death/retransplant

Variables	Hazard Ratio	95% CI	*p* -value
Pleural effusion	5.06	1.57-16.27	0.007
Restrictive defects	1.27	0.45-3.54	0.650
Obstructive defects	1.98	0.69-5.74	0.207
Diaphragmatic elevation	1.27	0.47-3.40	0.641

Abbreviations: CI, confidence intervals; CLAD, chronic lung allograft dysfunction; RAS, restrictive allograft syndrome; RLO, RAS-like opacities.

## Discussion

This study aimed to determine whether restrictive ventilatory defects stratify prognosis in non-RLO CLAD cases. Additionally, we examined radiographic findings associated with restrictive defects and assessed their relationship with clinical outcomes. Our multivariable analysis found a trend toward shorter overall survival in non-RLO CLAD patients with restrictive ventilatory defects. Importantly, when accounting for retransplantation as a competing event using Fine-Gray regression, this association reached statistical significance, confirming that restrictive ventilatory defects are independently associated with increased cumulative incidence of death. Furthermore, pleural effusion emerged as an independent prognostic factor in the non-RLO CLAD group, with significantly shorter overall survival observed in patients with this radiographic finding.

Undefined and unclassified phenotypes represented 41% of CLAD cases in our cohort, predominantly without RLO (97.1%), and showed no significant prognostic differences from BOS. This finding is clinically important because these emerging phenotypes, combined with BOS, constitute the majority (73.5%) of CLAD cases. While the current ISHLT classification relies primarily on obstructive parameters to categorize these phenotypes, our data suggest that restrictive ventilatory defects, rather than obstructive defects, may provide additional prognostic value within this non-RLO CLAD population. This challenges the assumption that all non-RLO CLAD cases follow a uniformly favorable BOS-like prognosis.^10^

The prognostic impact of restrictive ventilatory defects in non-RLO CLAD raises important mechanistic questions. Previous studies attributed restrictive physiology primarily to immunologically-mediated interstitial changes and pleural thickening seen in RAS.^6^, ^7^ However, our observation suggests that alternative non-parenchymal mechanisms may be operative in the absence of RLO. We therefore examined pleural effusion and diaphragmatic elevation, which are established causes of restrictive physiology,^11^, ^12^ as potential non-parenchymal contributors in this population. In our cohort, pleural effusion was observed in approximately 20% and diaphragmatic elevation in approximately half of non-RLO CLAD patients, with both findings predominantly present from baseline rather than developing acutely at the time of CLAD diagnosis.

These findings may be interpreted in the context of baseline lung allograft dysfunction (BLAD), a concept defined as failure to achieve normal lung function after lung transplantation. Recent evidence suggests that BLAD results from both parenchymal and extraparenchymal factors, including pleural effusion, chest wall restriction, and obesity, which can contribute to restrictive ventilatory defects and has been associated with increased mortality after lung transplantation.^24^

While neither pleural effusion nor diaphragmatic elevation showed statistically significant associations with restrictive ventilatory defects in our cohort, pleural effusion demonstrated a trend toward this association. Given that Darley et al. observed a higher prevalence of pleural effusions in BLAD patients,^25^ this raises the possibility that some restrictive non-RLO CLAD cases may represent a phenotypic extension of BLAD mediated by extraparenchymal mechanisms, suggesting pre-existing vulnerabilities rather than acute processes such as rejection or infection. The lack of association between diaphragmatic elevation and restrictive defects may reflect compensatory mechanisms by the accessory respiratory muscles.^26^

Regarding prognostic implications, both findings aligned with prior literature: pleural effusion emerged as a significant independent predictor of mortality, consistent with recent evidence,^27^ while diaphragmatic elevation showed no prognostic association.^17^ The significant association between pleural effusion and mortality, combined with its trend toward association with restrictive patterns, supports the concept that certain extraparenchymal factors contribute to a phenotype of vulnerability in lung transplant recipients.

These observations suggest that restrictive non-RLO CLAD may, in part, reflect BLAD-like vulnerability mediated by extraparenchymal mechanisms, potentially reducing tolerance to acute insults such as rejection or infection. However, it is important to note that we could not formally assess BLAD due to the unavailability of complete pulmonary function data required for its diagnosis. Prospective multicenter studies with comprehensive pulmonary function assessment are warranted to clarify the relationship between BLAD and non-RLO CLAD phenotypes.

Given the potential overlap between restrictive ventilatory defects and these radiographic findings, we conducted a multivariate Cox-hazard analysis to disentangle their independent prognostic contributions. In this comprehensive model, only pleural effusion remained significantly associated with poor outcomes. Notably, the association between restrictive ventilatory defects and survival was substantially attenuated in this model. This attenuation occurred despite the absence of substantial multicollinearity, suggesting that pleural effusion represents a stronger or more direct prognostic determinant than restrictive ventilatory defects.

This study has several limitations. First, this study is based on a single cohort with limited cases, requiring external validation in multicenter cohorts before informing clinical practice. Second, FVC was used as a surrogate for TLC,^3^, ^28^ potentially causing misclassification since BOS can also show FVC decline.^5^ Therefore, some cases that should have been diagnosed as BOS may have been misclassified as an undefined phenotype characterized by restrictive ventilatory defects. Third, the rarity of retransplantation in the non-RLO cohort (1 patient) may have limited the statistical power of the Fine-Gray competing risk analysis. However, the consistency of findings between the Cox regression and Fine-Gray models supports the robustness of our conclusions regarding the prognostic significance of restrictive ventilatory defects. Fourth, several potential confounders were unavailable in our cohort, including cytomegalovirus donor-recipient match status and primary graft dysfunction grade, both of which have been associated with post-transplant outcomes.^29^, ^30^ The absence of these variables may have introduced residual confounding in our survival analyses. However, prior studies have demonstrated that age and primary disease capture variations in immune competence, infection susceptibility, and comorbidity burden^19^, ^20^; therefore, adjustment for these variables helps partially account for unmeasured confounding from these interrelated factors. Fifth, pleural effusions were classified as present or absent, without severity grading, as most effusions were small in volume, limiting the utility of more granular classification. Finally, CT imaging at CLAD diagnosis was obtained at clinicians' discretion rather than by protocol, introducing selection bias as CT was more likely to be performed when abnormalities were noted on CXR. Consequently, CT scans were not available in some cases: 16 of 83 patients (19.3%) were assessed for RLO by CXR alone at CLAD diagnosis. Similarly, pleural effusion assessment at baseline, CLAD diagnosis, and follow-up also relied on CXR in some patients. Therefore, subtle RLO and pleural effusions may have been missed in cases evaluated only by CXR, potentially leading to underestimation of these findings. This potential selection bias introduced by the discretionary use of CT imaging might limit the generalizability of our findings.

In conclusion, our study demonstrates that restrictive ventilatory defects can stratify prognosis among non-RLO CLAD patients, showing a significantly increased cumulative incidence of death when accounting for retransplantation as a competing event. This challenges the assumption that all non-RLO CLAD cases follow a favorable BOS-like prognosis, revealing heterogeneity within this population. Pleural effusion independently predicted poor outcomes and may contribute to restrictive physiology in BLAD-like phenotypes. These findings underscore the importance of comprehensive clinical and radiological evaluation in non-RLO CLAD management. Future multicenter studies are needed to validate these associations and elucidate underlying mechanisms.

## Data Availability Statement

The data that support the findings of this study are available from the corresponding author upon reasonable request, subject to institutional review board approval and patient privacy restrictions.

## Funding

The authors received no funding for this work.

## CRediT authorship contribution statement

Conceptualization: T.F., H.H.; Methodology: T.F., H.H.; Formal analysis: T.F., Y.L., D.C.C.; Investigation: T.F., Y.N., S.T., M.N., H.H; Resources Data: T.F., S.T., M.N.; Data curation: T.F., S.T., M.N.; Writing - original draft: T.F.; Writing - review and editing: T.F., Y.N., Y.K., S.T., S.M.G., T.J., Y.L., D.C.C., H.O., L.S., M.N., H.H.; Visualization: T.F., Y.K., H.H.; Supervision: H.H.

## Disclosure statement

T.J. reports lecture fees from AstraZeneca, Boehringer Ingelheim, KYORIN Pharmaceutical Co, Ltd. Y.L. has received funding from an NIH (NCI) grant (R01CA249096 and R01CA269398). D.C.C. has received funding from an NIH (NCI) grant (U01-CA209414). L.S. has received funding from Genentech and Bristol Myers Squibb, and consulting fees from Genentech, AstraZeneca, and Lilly. M.N. has received a research grant to the institution from Canon Medical Systems and Konica Minolta Inc. H.H. is supported by grants NIH/NCI R01CA203636, NIH/NCI U01CA209414, NIH/NHLBI R01HL111024, NIH/NHLBI R01HL135142, and NIH/NHLBI R01HL130974, holds a provisional US patent application with the serial number 63/610,842, and has received a research grant to the institution from Canon Medical Systems. The remaining authors have no relevant disclosures.

## Declaration of Competing Interest

The authors declare that they have no known competing financial interests or personal relationships that could have appeared to influence the work reported in this paper.

## Footnotes

Appendix A Supplemental data associated with this article can be found in the online version at doi:10.1016/j.jhlto.2025.100445.
