# Supplementary material for: Prognostic impact of restrictive ventilatory defects in chronic lung allograft dysfunction without restrictive allograft syndrome-like opacities: Stratification of emerging undefined and unclassified phenotypes
Source: JHLT Open. 2025 Nov 24;11:100445. doi: 10.1016/j.jhlto.2025.100445 (PMC12757451; doi:10.1016/j.jhlto.2025.100445)
Supplement: Supplementary file 1 — Supplemental material [file mmc1.docx]

**Supplemental Table 1** Baseline characteristics of included and excluded patients

| Characteristics | Included | Excluded | *p* -value |
| --- | --- | --- | --- |
| Number of patients, n (%) | 241 (83.4) | 48 (16.6) |  |
| Age at Tx, years, median (IQR) | 58.0 (49.9-64.5) | 54.4 (30.0-61.9) | 0.012 |
| Gender male, n (%) | 134 (55.6) | 26 (54.2) | 0.981 |
| Primary disease, n (%) |  |  | 0.045 |
| Pulmonary fibrosis | 126 (52.3) | 23 (47.9) |  |
| COPD/emphysema | 58 (24.1) | 7 (14.6) |  |
| Cystic fibrosis | 36 (14.9) | 15 (31.2) |  |
| Other | 21 (8.7) | 3 (6.2) |  |

Abbreviations: COPD, chronic obstructive pulmonary disease; IQR, interquartile range; Tx, transplant.
